# Bedtime at Nana and Pop’s House

**DOI:** 10.3201/eid1405.52008

**Published:** 2008-05

**Authors:** Stan Shuman

Requires a hug and a kiss
From the two year old
In Mickey Mouse pajamas,
Climbing on my lap,
Interrupting the crime-news on T.V.,
Smack! a kiss on the left ear,
Smack! a kiss on the right,
"Eye, eye," the imp insists,
(Thank goodness for eyeglasses)
"Nose, nose," comes the next command.
I panic (what to do?!)
This adorable, cute, bright, affectionate kid,
My own grandchild,
Heading for the lips, now!
(What a strange ritual
The young parents have invented)
All I can think of, are GERMS:
Giardia, Hemophilus, E. coli,
Strep, Staph, and Pneumo,
A host of enterorespiratory viruses
Multiplying on this adorable child’s pink
Mucous membranes, fingertips,
His droplets and aerosols a sea of microbes.
I suddenly thrust him
At arm’s length, crown him
With a kiss on the curls
Of the cranium, blo
A few more long-distanc
Kisses as I hand him
To his mother
(Before any more infestation
can occur).
I return to the gloomy T.V.,
Wondering what the incubation
Periods are for the most likely
Forms of gastroenteritis, hepatitis,
Pink eye, U.R.I. and
Bronchopneumonia.
How fortunate the non-medical
Parents and co-grandparents,
Who hug, hug; kiss, kiss
Without worry or care!

